# Inhibitory effect of a novel Curcumin derivative DMC-HA on keloid fibroblasts

**DOI:** 10.18632/aging.205487

**Published:** 2024-01-26

**Authors:** Liang Hu, Zhicheng Bao

**Affiliations:** 1 Department of Burns and Plastic Surgery, Kunshan Hospital Affiliated to Jiangsu University, Kunshan 215300, Jiangsu, China; 2 Department of Rehabilitation Medicine, Kunshan Hospital Affiliated to Jiangsu University, Kunshan 215300, Jiangsu, China

**Keywords:** Curcumin, keloid, fibroblast, proliferation, apoptosis

## Abstract

Keloids pose a significant dermatological challenge, marked by abnormal fibroblast proliferation and excessive collagen deposition in response to skin injury or trauma. In the present study, we introduce DMC-HA, a derivative of Curcumin, as a promising candidate for keloid treatment. DMC-HA is poised to provide superior therapeutic benefits compared to Curcumin due to its structural modifications. Examining the comparative effects of DMC-HA and Curcumin on keloid fibroblasts can offer insights into their potential as therapeutic agents and the underlying mechanisms in keloid pathogenesis. In our study, CCK-8 experiments revealed that, at equivalent concentrations, DMC-HA demonstrated greater efficacy in inhibiting the proliferation of keloid fibroblasts compared to Curcumin. Flow cytometry analysis indicated that DMC-HA induced fibroblast apoptosis more significantly than Curcumin at the same concentration. Further data demonstrated that DMC-HA notably increased the production of reactive oxygen species (ROS), upregulated the expression levels of Bax, cleaved PARP, and cleaved Caspase-3. Interestingly, the impact of DMC-HA was reversed upon the application of the antioxidant NAC. Additionally, DMC-HA could suppress IL-6-induced increased expression of p-STAT3. Collectively, our findings suggest that DMC-HA is more effective than Curcumin in inhibiting the proliferation of keloid fibroblasts. The underlying mechanism of its action appears to be associated with the augmentation of ROS induction and the concurrent inhibition of STAT3 activation.

## INTRODUCTION

Keloids, also known as keloid scars, pose a dermatological challenge characterized by the abnormal formation of scar tissue exhibiting excessive collagen overgrowth, resulting in raised, often painful, and aesthetically displeasing skin lesions [1]. These scars arise from the wound healing process, wherein fibroblasts play a crucial role in collagen production, ultimately forming scar tissue. However, in the case of keloids, there is a disruption in the delicate balance between collagen production and degradation, leading to an undue accumulation of scar tissue [2]. This overproduction of collagen manifests in the characteristic features of keloids, including redness, a raised and hardened appearance, and a tendency to become more prominent over time [3]. Additionally, individuals affected by keloids often endure discomfort, itching, a sense of tightness, and challenges in joint mobility, rendering the condition not only an aesthetic concern but also a source of physical discomfort [4]. While several methods exist for treating keloid scars, such as pressure therapy, silicone gel application, laser therapy, surgical excision, and pharmacological interventions, effectively managing keloids remains a clinical challenge due to limitations associated with these treatments [5]. This underscores the need for continuous research to identify more potent therapeutic options.

Recent studies have investigated the potential therapeutic role of Curcumin, a natural polyphenolic compound derived from turmeric, in keloid treatment [6]. Curcumin has demonstrated anti-inflammatory, antioxidant, and anti-tumor properties [7, 8], making it an attractive candidate for keloid therapy. However, the effectiveness of Curcumin in keloid treatment has shown limitations, primarily due to factors such as low bioavailability, stability issues, and variable inhibition rates [9]. In response to these limitations, the present study explores the application of a novel Curcumin derivative known as DMC-HA (Dimethyl Curcumin-hyaluronic acid conjugate), which involves the conjugation of dimethyl Curcumin with hyaluronic acid (HA).

DMC-HA is designed to overcome the challenges associated with Curcumin. Hyaluronic acid, a naturally occurring polysaccharide with remarkable biocompatibility and biodegradability, has been used in diverse biological contexts due to its lubricating, moisturizing, and cell proliferation regulatory properties [10, 11]. Combining dimethyl Curcumin with hyaluronic acid in the form of the DMC-HA conjugate offers several advantages. These include enhanced bioavailability, improved stability through protection against light, heat, and oxygen, targeted delivery to inflammatory tissues, and controlled release of the active compound [12, 13]. The aim of DMC-HA is to provide a more effective solution for keloid treatment by addressing the limitations associated with Curcumin.

In this study, we explore the comparative effects of DMC-HA and Curcumin on keloid fibroblasts, aiming to assess the potential of DMC-HA in inhibiting keloid proliferation and promoting apoptosis, while also elucidating the underlying molecular mechanisms responsible for its effects.

## RESULTS

### DMC-HA is more effective than Curcumin in inhibiting keloid fibroblast proliferation

**Figure 1:**
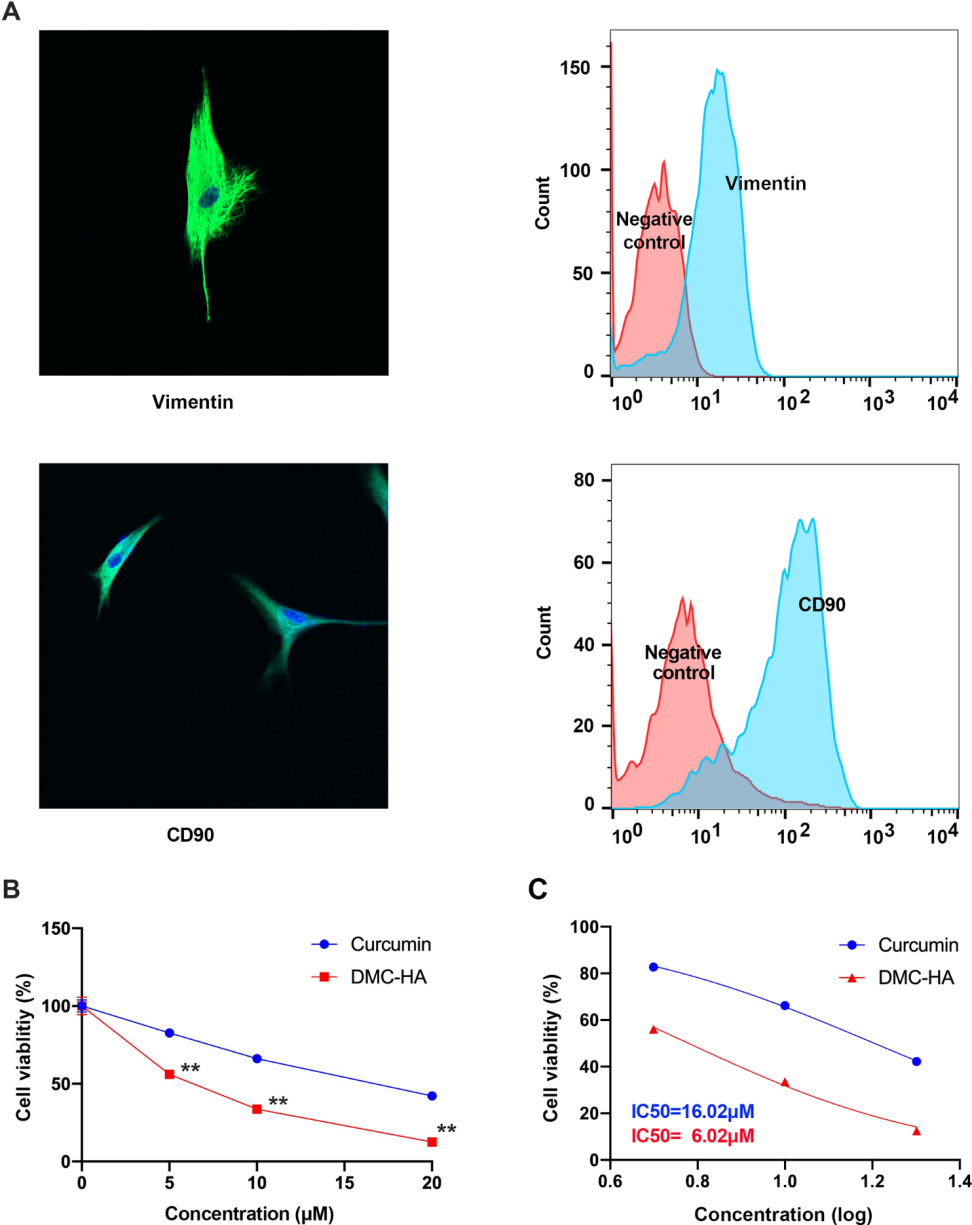
**Effects of DMC-HA and Curcumin on the cell viability of primary keloid fibroblasts.** (**A**) Immunocytochemistry and flow cytometry revealed primary keloid fibroblasts expressed Vimentin and CD90 which were major markers of fibroblast; (**B**) Cell viability of primary keloid fibroblasts was assessed using the CCK-8 assay following treatment with DMC-HA and Curcumin; (**C**) IC50 of DMC-HA and Curcumin on primary keloid fibroblasts.

Vimentin and CD90 serve as valuable markers in differentiating fibroblasts and exploring their roles in diverse biological processes. In our study, we performed *in situ* immunohistochemical staining for Vimentin and CD90 on primary cultured keloid fibroblasts. As depicted in Figure 1A, the immunohistochemical staining revealed robust expression of Vimentin and CD90 primarily within the cytoplasm of keloid fibroblasts, a finding further corroborated by flow cytometry analysis.

Subsequently, we subjected these keloid fibroblasts to DMC-HA and Curcumin treatments at varying concentrations (0, 5, 10, and 20 μM) for a duration of 72 h. The CCK-8 assay results, displayed in Figure 1B, indicate that both drugs demonstrated the ability to inhibit keloid fibroblast proliferation, with the inhibitory effect intensifying as drug concentration increased. However, when comparing equivalent concentration levels, it became evident that DMC-HA exhibited a significantly superior inhibitory effect on cell proliferation compared to Curcumin (P<0.05). Further analysis revealed that the IC50 value for DMC-HA was 6.02 μM, a significant improvement over Curcumin, which had an IC50 value of 16.02 μM (Figure 1C).

### DMC-HA is more effective than Curcumin in inducing apoptosis of keloid fibroblasts

**Figure 2:**
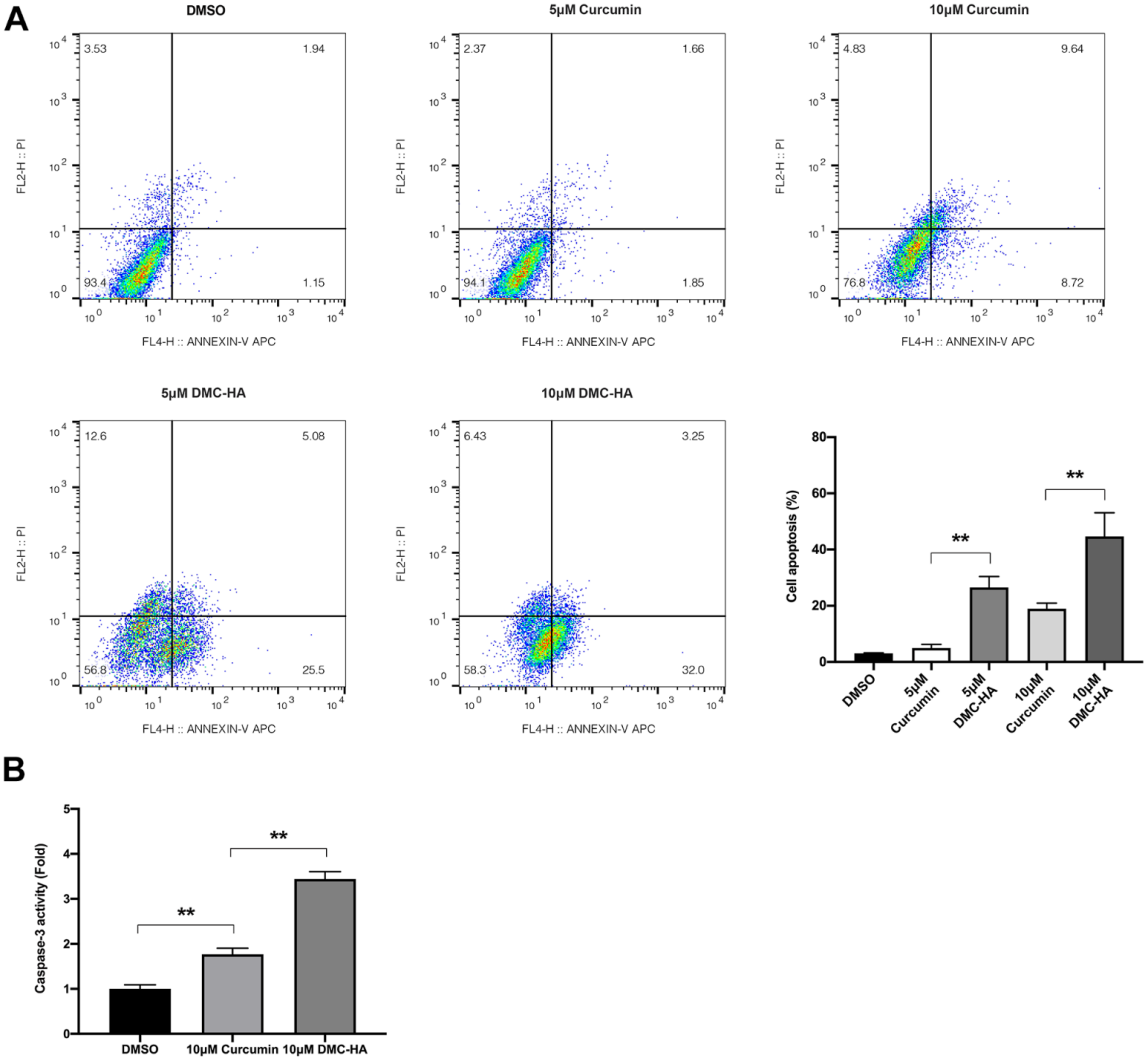
**Effects of DMC-HA and Curcumin on the cell apoptosis of primary keloid fibroblasts.** (**A**) Cell apoptosis of primary keloid fibroblasts was assessed using the flow cytometry assay following treatment with Curcumin or DMC-HA; (**B**) The caspase-3 activity of primary keloid fibroblasts was detected after Curcumin or DMC-HA treatment.

Following the determination of the IC50 value through the aforementioned CCK-8 assay, we proceeded to assess the impact of DMC-HA on keloid fibroblast apoptosis. Keloid fibroblasts were subjected to treatments with varying concentrations of 5 and 10 μM of DMC-HA or Curcumin for a duration of 24 h, after which apoptosis was quantified using flow cytometry. As depicted in Figure 2A, the flow cytometric analysis consistently demonstrated a significantly higher rate of apoptosis in keloid fibroblasts when exposed to DMC-HA compared to Curcumin, across equivalent concentrations of 5 and 10 μM (P<0.05). Notably, Curcumin concentrations below 5 μM failed to induce substantial apoptosis in keloid fibroblasts (P>0.05).

To further support these findings, we conducted an assessment of caspase-3 activity, a crucial marker of apoptosis. The results were striking, indicating that 10 μM DMC-HA led to a significant 1.68-fold increase in caspase-3 activity when compared to the same concentration of Curcumin (Figure 2B). These results underscore the remarkable apoptotic-inducing potential of DMC-HA, particularly at the 10 μM concentration.

### DMC-HA induces keloid fibroblast apoptosis through ROS

To investigate the impact of DMC-BH on ROS production, cells were pre-exposed to 5 and 10 μM DMC-BH for 24 h. In comparison to the control group, flow cytometry analysis revealed a noteworthy increase in ROS levels following DMC-HA pretreatment (P<0.05) (Figure 3A). Subsequently, Western blot analysis, conducted after a 24-hour DMC-HA intervention, demonstrated a substantial upregulation in intracellular mitochondrial apoptosis-related proteins, including cleaved caspase-3 (c.Casp.-3), cleaved PARP (c.PARP), Bax, and cytochrome C (Cyto C) (P<0.05) (Figure 3B).

**Figure 3:**
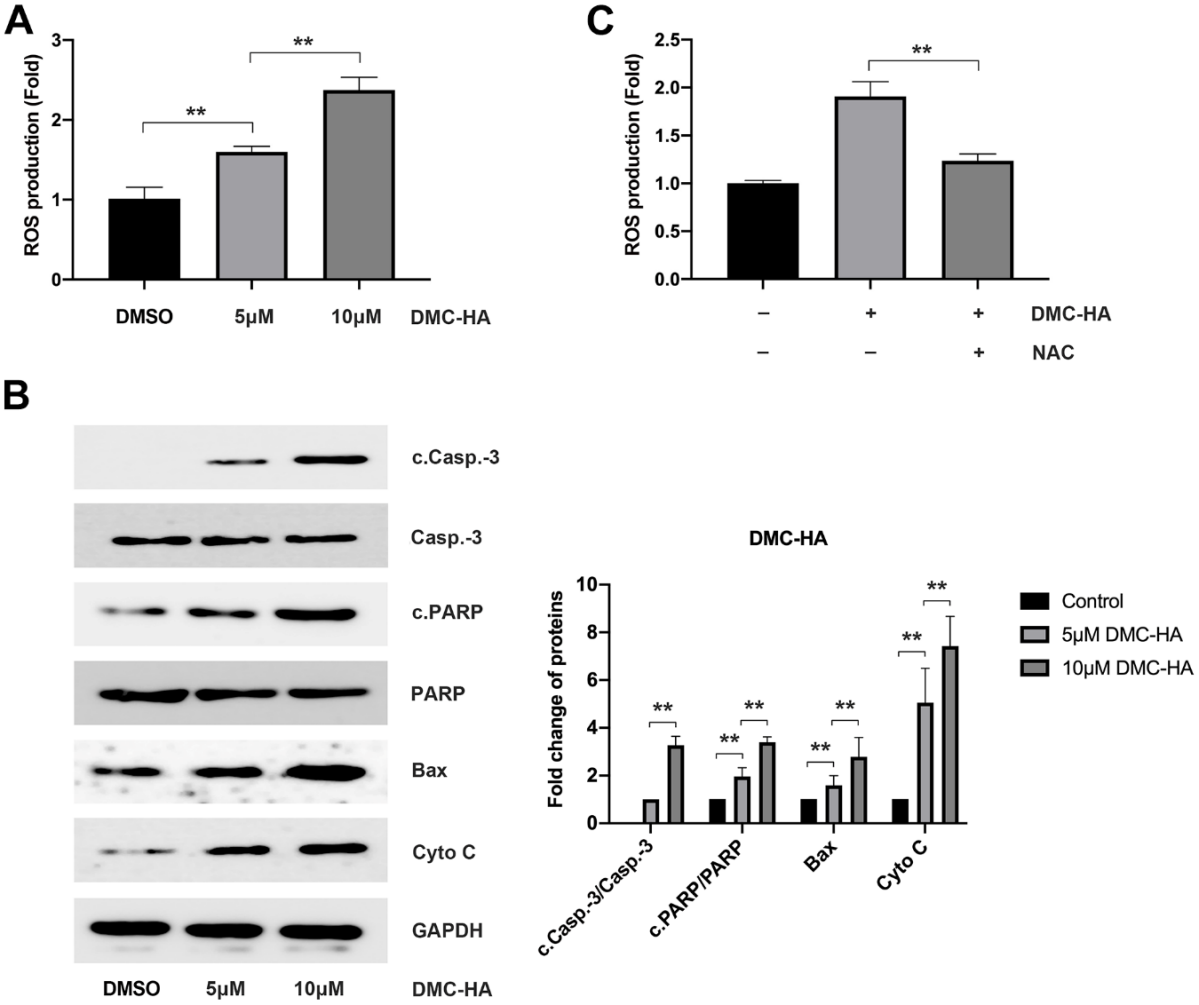
**Effects of DMC-HA on ROS production.** (**A**) The impact of DMC-HA on ROS generation; (**B**) The effect of DMC-HA on the expression of c.Casp.-3, c.PARP, Bax, and Cyto C proteins; (**C**) NAC administration during DMC-HA intervention and its effect on ROS generation.

**Figure 3:**
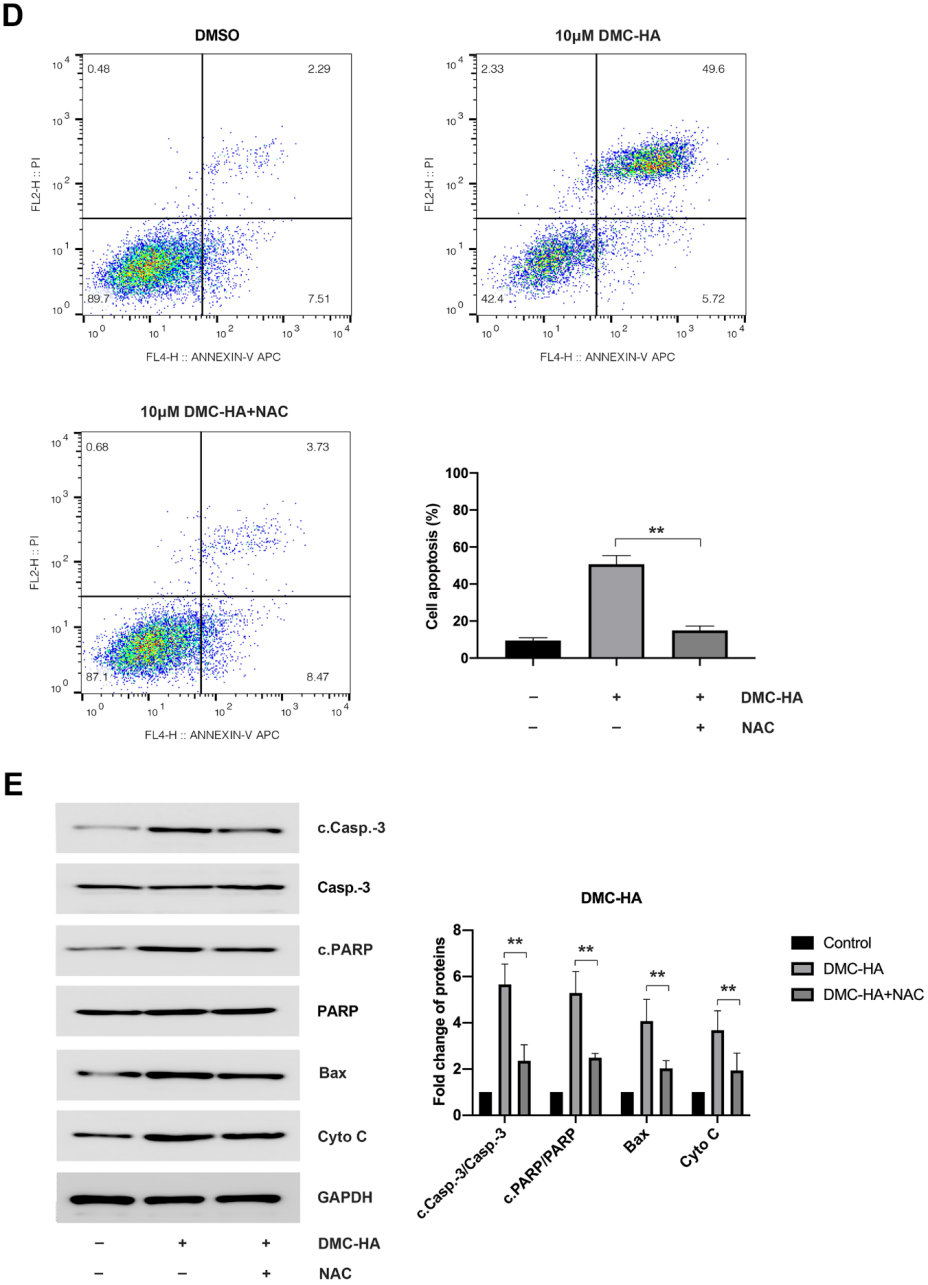
**Effects of DMC-HA on ROS production.** (**D**) NAC-mediated attenuation of cell apoptosis induced by DMC-HA; (**E**) NAC-induced alterations in the expression of c.Casp.-3, cleaved PARP, Bax, and Cyto C proteins in the presence of DMC-HA.

Upon the introduction of the ROS scavenger, NAC, a significant reduction in intracellular ROS levels induced by 10 μM DMC-HA in keloid fibroblasts was observed compared to the control group (Figure 3C). Additionally, pre-treatment with NAC ameliorated the apoptosis induced by DMC-HA (Figure 3D). Consistently, Western blot analysis revealed that pretreatment with NAC resulted in a reduction in the expression of c.Casp.-3, c.PARP, Bax, and Cyto C proteins, which had been upregulated by DMC-HA (Figure 3E).

### DMC-HA inhibits the STAT3 pathway in keloid fibroblasts

We proceeded to investigate the potential involvement of the STAT3 signaling pathway in the inhibitory effect of DMC-HA on keloid fibroblasts. Previous studies have indicated that *in vitro* cultured keloid fibroblasts, similar to their normal fibroblast counterparts, either do not express phosphorylated STAT3 (p-STAT3) or express it at very low levels. However, the area surrounding keloid lesions exhibits heightened expression of IL-6, which contributes to keloid formation by inducing STAT3 phosphorylation in fibroblasts.

**Figure 4:**
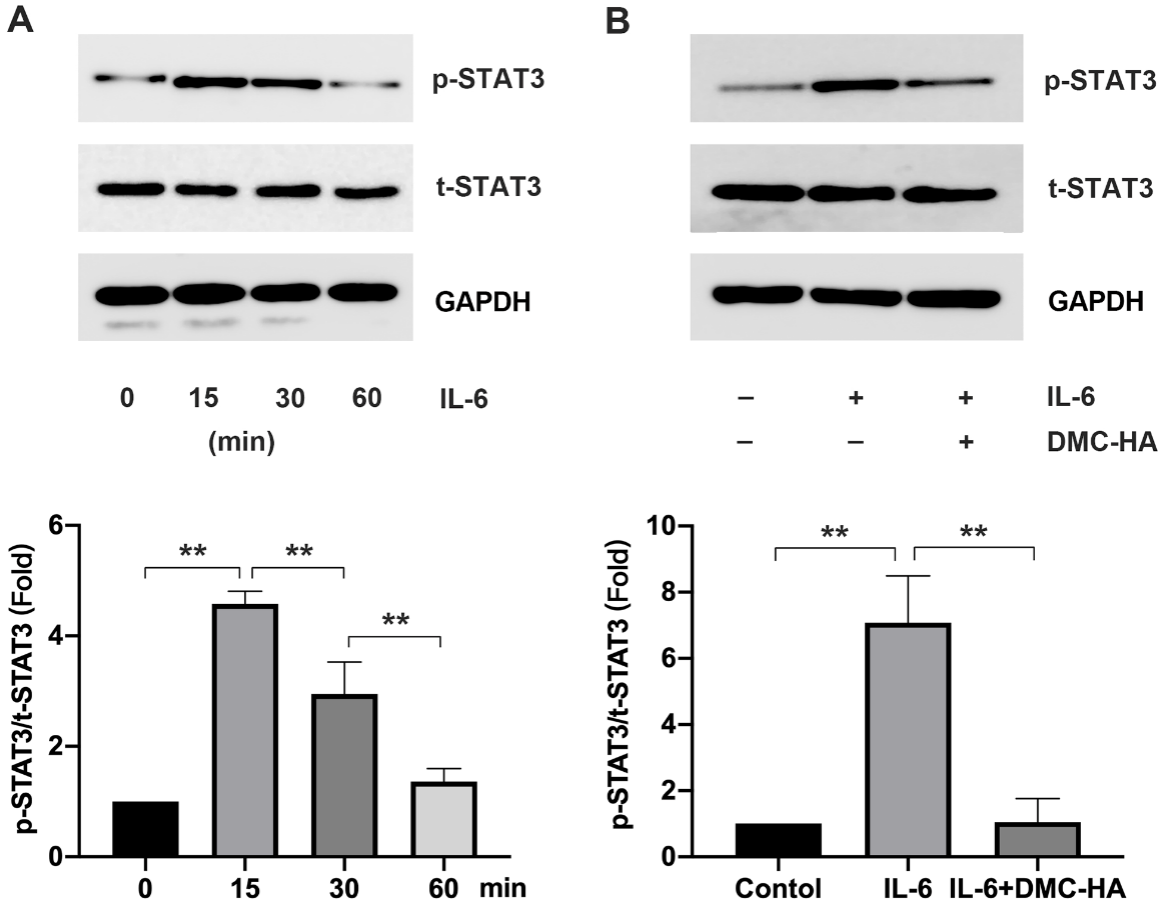
**Effects of DMC-HA on STAT3 pathway.** (**A**) p-STAT3 expression after exogenous IL-6 intervention; (**B**) p-STAT3 expression after exogenous IL-6 combined with DMC-HA intervention.

Consequently, upon the introduction of exogenous IL-6 to keloid fibroblasts, we observed a substantial increase in p-STAT3 levels, with the peak occurring at 15 min post-intervention (Figure 4A). Subsequent addition of DMC-HA resulted in a reduction in p-STAT3 levels induced by exogenous IL-6 in keloid fibroblasts, as evidenced by Western blot analysis (P<0.05) (Figure 4B). These findings suggest that DMC-HA may manifest its anti-scar effects through the inhibition of the STAT3 signaling pathway.

## DISCUSSION

Curcumin, a natural polyphenolic compound primarily derived from turmeric (Curcuma longa) rhizomes [14], has garnered considerable attention in recent years due to its wide-ranging biological activities, including antioxidant, anti-inflammatory, and anti-tumor effects [15]. Interestingly, recent research has explored the potential of Curcumin in keloid treatment. Curcumin has been shown to inhibit the production of inflammation-related cytokines such as tumor necrosis factor-α (TNF-α), interleukin-1β (IL-1β), and interleukin-6 (IL-6) [16–18], all of which play pivotal roles in keloid formation. Additionally, Curcumin has demonstrated the ability to curb fibroblast proliferation, the primary cell type implicated in keloid development [19, 20]. Despite its significant biological activities in inflammation regulation, antioxidant effects, and scar mitigation, Curcumin does have limitations, including low bioavailability, stability, and inhibition rates [21].

To address these limitations, we introduced DMC-HA, a conjugate consisting of the Curcumin derivative dimethyl Curcumin (DMC) and hyaluronic acid (HA), linked through covalent bonds. Dimethyl Curcumin, a derivative of Curcumin, offers enhanced bioavailability and stability [22]. Hyaluronic acid, a naturally occurring polysaccharide known for its exceptional biocompatibility, biodegradability, and gel-forming properties, is widely distributed in human connective tissues, the vitreous humor of the eye, and joint synovial fluid. It serves various biological functions, including lubrication, moisturization, and the regulation of cell proliferation [23]. The synthesis and preparation process of DMC-HA, referred to as Dimethyl Curcumin-Hyaluronic Acid, is comprehensively described by Wang et al. [24]. This process involves the covalent linkage of the DMC and HA. This innovative approach was employed to overcome the limitations associated with Curcumin, specifically related to issues of bioavailability, stability, and targeted drug delivery.

In this study, we conducted a comparative analysis of the effects of DMC-HA and Curcumin on keloid fibroblasts. The experimental results obtained from the CCK-8 assay and flow cytometry analysis demonstrate that, at equivalent concentrations, DMC-HA is more effective in inhibiting keloid fibroblast proliferation and inducing apoptosis compared to Curcumin. Furthermore, as the drug concentration increases, DMC-HA exhibited an increasingly pronounced anti-keloid fibroblast effect. Western blot analysis suggested that this inhibition of proliferation and induction of apoptosis may be associated with the activation of apoptosis-related signaling pathways. The mitochondrial apoptosis pathway plays a pivotal role in the regulation of programmed cell death, with reactive oxygen species (ROS) acting as dual-role molecules in cellular processes [25]. ROS comprise a group of oxygen-derived molecules, including superoxide anion (O2•−), hydrogen peroxide (H2O2), and hydroxyl radical (•OH), and they are produced as natural byproducts of mitochondrial respiration and various cellular metabolic processes [26]. These molecules play a multifaceted role in cellular physiology. On one hand, an appropriate and controlled level of ROS serves as vital cell signaling molecules, actively participating in the regulation of essential cellular processes, such as cell growth, differentiation, and apoptosis [27–29]. The modulation of ROS levels can, in turn, affect key intracellular signaling pathways, including those involved in cell proliferation and survival [30].

Previous research has affirmed Curcumin’s influence on ROS and the mitochondrial apoptosis pathways [31]. Given that DMC-HA is a derivative of Curcumin, we evaluated its impact on the ROS-dependent mitochondrial apoptosis pathway. Experimental results from the ROS assay demonstrated that DMC-HA effectively elevated ROS levels in keloid fibroblasts and enhanced Caspase-3 activity within mitochondria. Western blot analysis further revealed that DMC-HA promoted the aggregation of mitochondrial channel proteins, such as Bax, leading to the release of mitochondrial Cyto C, consequently increasing the activity of the apoptosis execution enzyme, cleaved caspase-3. ADP-ribose polymerase (PARP), an essential DNA repair enzyme responsible for mending single- and double-strand DNA breaks caused by damage [32], also saw increased expression following DMC-HA intervention. Notably, when the ROS scavenger NAC was applied, the effects of DMC-HA were significantly attenuated. Hence, DMC-HA effectively promotes cell apoptosis by modulating the ROS-dependent mitochondrial apoptosis pathway and triggering Caspase-3-mediated PARP cleavage.

STAT3 can directly or indirectly regulate mitochondrial function. Research has uncovered STAT3’s ability to translocate into mitochondria, interact with mitochondrial proteins, and affect mitochondrial membrane potential, ROS production, and the activation of the mitochondrial apoptosis pathway [33]. Studies have also established the critical role of IL-6 in keloid formation [34]. During wound healing, trauma initiates an inflammatory response, leading to the release of substantial amounts of IL-6. While IL-6 can accelerate wound healing by promoting an inflammatory response, it can also stimulate fibroblasts to produce collagen excessively, contributing to scar proliferation and keloid formation when secreted excessively. Previous studies have demonstrated that IL-6 stimulates the phosphorylation of pY705 STAT3 in keloid fibroblasts to a significantly higher degree than in normal fibroblasts [35]. In our study, p-STAT3 expression significantly increased in keloid fibroblasts following exogenous IL-6 stimulation, but this elevated p-STAT3 was significantly suppressed by DMC-HA intervention. This suggests that DMC-HA has the potential to inhibit keloid formation by mitigating STAT3 phosphorylation.

While our study shows promise, it has some limitations. It primarily relies on *in vitro* experiments with keloid fibroblasts, which may not fully replicate *in vivo* conditions. The absence of *in vivo* experiments using animal models or human subjects is a notable limitation. Furthermore, the study has a relatively short 72-hour observation period for *in vitro* experiments, and the concentration-dependent effects are not thoroughly explored. Additionally, the study lacks clinical data, leaving practical application and real-world patient outcomes unaddressed. Future research should consider these limitations to provide a more comprehensive assessment of DMC-HA’s potential for keloid treatment.

In conclusion, DMC-HA elevates intracellular ROS levels, inhibits STAT3 phosphorylation, and activates the mitochondrial apoptosis pathway, ultimately inhibiting keloid fibroblast proliferation and promoting apoptosis, thereby suppressing keloid formation.

## MATERIALS AND METHODS

### Materials

Keloid tissue specimens were acquired from patients undergoing surgical removal. No interventions or treatments were administered to the patients before the surgical procedures, ensuring the preservation of comprehensive clinical data. All aspects of this research were conducted in compliance with the guidelines and regulations established by the Ethics Committee of Affiliated Kunshan Hospital of Jiangsu University (2023-03-058-K01). Moreover, informed consent was diligently obtained from all participating patients, emphasizing the ethical integrity of the study.

### Primary keloid fibroblast culture

Under strict sterile conditions, a small piece of tissue is excised from the patient’s keloid site. The excised scar tissue is then transferred to a sterile petri dish and subjected to multiple rinses with physiological saline or PBS (#KGB5001, KeyGEN Biotechnology, Nanjing, China) in order to eliminate blood and impurities. Subsequently, sterile scissors and forceps are used to cut the tissue into approximately 1mm^3^-sized pieces. After the addition of 0.25% trypsin/EDTA (#BL501A, Biosharp, Anhui, China) for digestion for 30 min, the tissue was gently agitated in a constant-temperature water bath set at 37° C for 1-2 h to aid in the release of fibroblasts from the tissue matrix. After the digestion process, complete culture medium (#KGM12800S-500, KeyGEN Biotechnology, Nanjing, China) was added to halt the reaction. Subsequently, the suspension was centrifuged at 1500 rpm for 5 min collect the cells. The supernatant was carefully decanted, and the cells were then resuspended in high-glucose Dulbecco’s Modified Eagle Medium (DMEM) containing 10% fetal bovine serum (#12484028, Gibco BRL, Grand Island, NY, USA). These cells were maintained in a controlled incubator at 37° C with a 5% CO_2_ environment. The initial medium change occurred after 3 days, followed by subsequent changes every 3 days. Routine passage and digestion were carried out when the cell confluence reached 70%-80%. Cells from passages 3 to 6 were used in the experimental procedures.

### Immunofluorescence staining

Keloid fibroblasts were initially cultured in a culture dish and allowed to adhere to the dish’s surface. Subsequently, the cells were fixed with 4% acetaldehyde, followed by two 5-minute PBS washes. To block non-specific binding, a 5% bovine serum albumin solution was applied for 30 min. A Vimentin (#AF1975, Beyotime Biotechnology, Shanghai, China) or CD90 (#AF1636, Beyotime Biotechnology, Shanghai, China) primary antibody (1:100) was added, and the cells were incubated for 1 hour, after which they were washed with PBS to remove any unbound primary antibodies. A FITC-labeled goat anti-rabbit IgG (#A0562, Beyotime Biotechnology, Shanghai, China) (1:500) solution was then added to the cells and allowed to incubate for 30 min. Following this, the cells were washed with PBS to remove unbound secondary antibodies. Next, the nuclear stain DAPI (#C1005, Beyotime Biotechnology, Shanghai, China) was applied as a counterstain, and a drop of the appropriate anti-fade agent, Husolone, was added to the glass cover slip. The cover slip was then placed over the cells and secured using a blocking agent. Subsequently, a confocal microscopy was employed to visualize the fluorescent signal within the cells and capture images.

### Flow cytometry for Vimentin and CD90 detection

Cultured cells were harvested and incubated with FITC-conjugated CD90 (#bsm-30105M-FITC, Bioss Biotechnology, Shanghai, China) and FITC-conjugated Vimentin (ab128507, Abcam, Cambridge, MA, USA) at 4° C for 30 min. After two washes, the cells were analyzed using a BD FACS Calibur cytometer. Flow cytometry data were processed using Flowjo software.

### CCK-8 method for detecting cell proliferation

Keloid fibroblasts were collected and cultured, and the cells were seeded into 96-well plates at a density of 5,000 cells per well. When the cells are attached and grown to 70-80% passage, different concentrations of 0, 5, 10, 20 μM DMC-HA and Curcumin were added to treat the cells, and continued to culture them in a 37° C, 5% CO_2_ incubator for 24, 48 or 72h to observe the effect of Curcumin on cell proliferation at different time points. After the treatment, 10 μL of CCK-8 solution (#C0037, Beyotime Biotechnology, Shanghai, China) was added to each well, and then continue to incubate the cells for 1-4 h in a 37° C, 5% CO_2_ incubator. An enzyme-linked immunosorbent assay (ELISA) reader was employed to measure the absorbance values at a wavelength of 450 nm for each well. Subsequently, perform data analysis and generate graphical representations depicting the correlation between absorbance values and the concentrations of DMC-HA and Curcumin.

### Flow cytometry for detecting cell apoptosis

Keloid fibroblasts were treated with different concentrations of DMC-HA and Curcumin, and the cells were collected at 24 h. Cells were centrifuged using serum-free medium and cell density was adjusted to 1x10^6 cells/mL. 100 μL of cell suspension was added to the flow cytometry tube. 5 μL of Annexin V-APC was added to each flow cytometry tube and incubated in the dark at room temperature for 15 min. 5 μL of PI dye was added to each flow cytometry tube and mix gently. Within 10 min after adding the PI dye, the cell apoptosis rate was analyzed using flow cytometry.

### ROS measurement

To assess intracellular ROS production in DMC-HA-treated cells, fluorescence-activated cell sorting (FACS) analysis was conducted. Cells were exposed to varying DMC-HA concentrations for 24 h, followed by staining with 5 μg/ml of 2’,7’-dichlorofluorescin diacetate (DCF-DA) for 30 min. Subsequently, the stained cells were analyzed using a Becton-Dickinson FACS Caliber flow cytometer (Becton-Dickinson, San Jose, CA, USA).

### Measurement of caspase 3 activity

The activity of Caspase 3 was assessed using a Caspase Activity Detection kit (Nanjing Kaiji Biotechnology, China) following the manufacturer’s instructions. Cell lysis was performed using the colorimetric buffers provided in the Caspase Activity Detection kit. Subsequently, proteins were pelleted by centrifugation at 12,000 × g for 10 min at 4° C and quantified using the BCA Protein assay kit (Nanjing Kaiji Biotechnology, China). Next, 100 μg of protein was incubated with 5 μl of the caspase 3 substrate (Ac-DEVD-pNA) in a 96-well plate. Caspase 3 activity was determined spectrophotometrically at 405 nm.

### Western blot analysis

After keloid fibroblasts were treated with different concentrations of DMC-HA for 24 h, the cells were collected and proteins were extracted using RIPA lysis buffer, and then the cell lysate was separated by centrifugation. Next, the protein concentration was determined using the BCA method. An equal amount of protein sample (such as 50 μg) was added to the SDS-PAGE gel and electrophorese at 140V voltage for 1.5h. The protein was transferred to the PVDF membrane and 300mA current was used for transfer for 1 h. After the transfer is completed, 5% skim milk was used to block the non-specific binding sites of the PVDF membrane, and then incubated with specific primary antibodies at 4° C overnight. The next day, the membrane is incubated with the corresponding secondary antibody, usually for 1-2 h at room temperature. Finally, signals were detected with enhanced chemiluminescence (ECL) reagents, and images were acquired using a chemiluminescence imaging system. Analyze protein expression levels and perform quantitative and statistical analysis of experimental results as needed.

### Statistical analysis

Data analysis was performed using SPSS 21.0 statistical software. In the comparison between multiple groups of data, one-way analysis of variance (ANOVA) was performed, while the t test was used for the comparison of two groups of experimental data. Set the test significance threshold α to 0.05.
